# Production of Feminized Seeds of High CBD *Cannabis sativa* L. by Manipulation of Sex Expression and Its Application to Breeding

**DOI:** 10.3389/fpls.2021.718092

**Published:** 2021-11-01

**Authors:** Marko Flajšman, Miha Slapnik, Jana Murovec

**Affiliations:** Department of Agronomy, Biotechnical Faculty, University of Ljubljana, Ljubljana, Slovenia

**Keywords:** *Cannabis sativa* L., sex manipulation, silver thiosulfate, cannabidiol, high CBD medical cannabis, feminized seed, cannabinoids

## Abstract

The use of the cannabis plant as a source of therapeutic compounds is gaining great importance since restrictions on its growth and use are gradually reduced throughout the world. Intensification of medical (drug type) cannabis production stimulated breeding activities aimed at developing new, improved cultivars with precisely defined, and stable cannabinoid profiles. The effects of several exogenous substances, known to be involved in sex expressions, such as silver thiosulfate (STS), gibberellic acid (GA), and colloidal silver, were analyzed in this study. Various concentrations were tested within 23 different treatments on two high cannabidiol (CBD) breeding populations. Our results showed that spraying whole plants with STS once is more efficient than the application of STS on shoot tips while spraying plants with 0.01% GA and intensive cutting is ineffective in stimulating the production of male flowers. Additionally, spraying whole plants with colloidal silver was also shown to be effective in the induction of male flowers on female plants, since it produced up to 379 male flowers per plant. The viability and fertility of the induced male flowers were confirmed by fluorescein diacetate (FDA) staining of pollen grains, *in vitro* and *in vivo* germination tests of pollen, counting the number of seeds developed after hybridization, and evaluating germination rates of developed seeds. Finally, one established protocol was implemented for crossing selected female plants. The cannabinoid profile of the progeny was compared with the profile of the parental population and an improvement in the biochemical profile of the breeding population was confirmed. The progeny had a higher and more uniform total CBD (tCBD) to total tetrahydrocannabinol (tTHC) ratio (up to 29.6; average 21.33 ± 0.39) compared with the original population (up to 18.8; average 7.83 ± 1.03). This is the first comprehensive report on the induction of fertile male flowers on female plants from dioecious medical cannabis (*Cannabis sativa* L.).

## Introduction

Cannabis (*Cannabis sativa* L.) naturally shows sexual dimorphism with a small proportion of monoecism. In the past, it was mostly cultivated for fiber and grain, but nowadays, the plant is gaining importance in the medicinal industry due to its production of unique cannabinoids (Andre et al., 2016). They are produced in the trichomes on flower bracts of female inflorescences (Small, 2015; Andre et al., 2016). Most pharmaceutically important cannabinoids are cannabidiol (CBD) and the psychoactive tetrahydrocannabinol (THC) (Δ-9-THC) (Freeman et al., 2019). The relative content (in % of dry weight) of the latter divides cannabis genotypes into two groups: (i) industrial cannabis, commonly known as hemp or fiber-type hemp (defined as containing < 0.2% THC by dry weight in Europe) and commonly grown as a field crop and (ii) medical cannabis, marijuana or drug type cannabis (with > 0.2% THC) (The European Commission, 2014), cultivated under strict legal restrictions.

Sex of *C. sativa* L. (2n = 20) is genetically determined by one pair of sex chromosomes X and Y, where male gender of dioecious plants is determined by heterogametic XY chromosomes, while dioecious female and monoecious or hermaphrodite plants exhibit homogametic chromosomes XX (Moliterni et al., 2004; van Bakel et al., 2011; Divashuk et al., 2014; Faux et al., 2014). The ratio of female to male flowers in a single monoecious cannabis plant is highly variable and ranges from predominantly male flowers to predominantly female flowers (Faux et al., 2014). Moreover, dioecious cannabis plants can produce flowers of the opposite sex as determined by their sex chromosomes (Moliterni et al., 2004). Due to instability of the sexual phenotypes across generations of XX plants, and the quantitative nature of sex expression, it was hypothesized that sex expression is a polygenic trait (Faux et al., 2013, 2014; Faux and Bertin, 2014). A first association mapping study of sex determination was performed in 2016 (Faux et al., 2016) on three biparental hemp populations (two dioecious and one monoecious) using 71 amplified fragment length polymorphism (AFLP) markers. It identified five quantitative trait loci (QTLs) associated with sex expressions that were putatively located on sex chromosomes. Recently, Petit and colleagues (Petit et al., 2020) published the results of a GWAS (Genome-Wide Association Study) analysis for characterization of the genetic architecture underpinning sex determination in hemp. They used a set of 600 K single-nucleotide polymorphism (SNP) markers on a panel of 123 hemp accessions (monoecious and dioecious), tested in three contrasting environments across Europe with contrasting photoperiod regimes. They identified two QTLs for sex determination across locations that contained transcription factors and genes involved in regulating the balance of phytohormones, especially auxins and gibberellic acid (GA). Two auxin response factor genes (*arf2* and *arf5*), *bZIP* transcription factor 16-like, and gene *gibberellic acid insensitive* (GAI) that codes for the DELLA RGL1-like (repressor of giberellic acid-like) protein were identified in QTL*Sex_det1* for sex determination. These genes are involved in the balance of the phytohormones auxins and gibberellic acid (GA), which are known to play an active role in the sex expression (male or female) in many crops, such as hemp or spinach. The lack of a complete genome sequence did not allow to map of the QTL*Sex_det1* in any specific chromosome (Petit et al., 2020).

The findings confirmed previous reports that several factors, like sex-determining genes, sex chromosomes, epigenetic control by DNA methylation, and microRNAs, and physiological regulation with phytohormones influence sex expression of predetermined cannabis plants (Galoch, 1978; Dellaporta and Calderon-Urrea, 1993; Hall et al., 2012; Punja and Holmes, 2020). Several studies with hormonal manipulation confirmed gender reversal in *C. sativa* L. and proved bipotency of sexually predetermined dioecious cannabis plants. It has been shown that gibberellins induce maleness in plants, while ethylene, cytokinins, and auxins stimulate the formation of female flowers on genetically male plants (Ainsworth, 2000). Galoch (1978) showed that indole-3-acetic acid (IAA), kinetin (up to 100 μg/plant), and ethylene-releasing compound ethrel (up to 500 μg/plant) enhanced the feminization of male plants. Abscisic acid (ABA) was completely ineffective in sexing both male and female hemp when used alone. GA3 (up to 100 μg/plant) promoted masculinization of female plants while having no effect on sex change in male plants. Similarly, Ram and Jaiswal (1972) earlier found that male plants showed no change in sex expression when treated with gibberellins (up to 100 μg/plant), but female plants developed male flowers with normal stamens and viable pollen grains. Besides, environmental factors such as temperature, photoperiod, light conditions, nutrient deficiency, and mechanical stresses (e.g., damages) can influence sex expression and induce monoecism (Ram and Sett, 1979). As reviewed in Truta et al. (2007) and Petit et al. (2020), the ratio of different phytohormones plays a crucial role in the sex expression of hemp. External treatment of GA to spinach, for example, affects the expression of the *GAI* gene, which is a transcription factor of the DELLA family. It is highly expressed in female inflorescences and acts as a repressor of the expression of B-*class* homeotic genes, which are masculinizing factors. B-*class* genes stimulate male organ formation and simultaneously suppress the development of female organs in the flowers (Petit et al., 2020).

Cannabis sex determination could be modified by applying exogenous growth regulators or chemicals, which can influence the ratio of endogenous hormones and hence the incidence of sex organs (Truta et al., 2007). Silver compounds such as silver nitrate (AgNO_3_) or silver thiosulfate (Ag_2_S_2_O_3_; STS) have been found to have masculine effects in many plant species, e.g., in *Coccinia grandis* (Devani et al., 2017), *Cucumis sativus* (Den Nijs and Visser, 1980), *Silene latifolia* (Law et al., 2002), *Cucumis melo* (Owens et al., 1980), and also *Cannabis sativa*. Ram and Sett (1982) applied 50, 100, and 150 μg of silver nitrate and 25, 50, and 100 μg of STS to shoot tips of female cannabis plants. Both silver compounds successfully evoked the formation of male flowers, but STS was more effective than AgNO_3_. 100 μg of STS caused the highest number of fully altered male flowers, which was significantly higher than the number of reduced male, intersexual, and female flowers. On the other hand, the treatment of shoot tip with 100 μg AgNO_3_ resulted in more than half the lower number of male flowers, with the highest amount of AgNO_3_ (150 μg) being ineffective in altering sex expression. Furthermore, pollen from all induced male flowers was viable *in vitro* and also successfully induced seed set. Lubell and Brand (2018) published the results of using 3 and 0.3 mM STS to induce male flowers in genetically female hemp plants of four strains. They sprayed three times at 7-day intervals and counted flowers (male and female) on terminal buds, not whole plants. They determined the percentage of male flowers to all flowers and the masculinization rate. The authors confirmed the successful induction of male flowers in hemp strains. Regarding the percentage of inflorescences with male flowers, their best two hemp strains yielded up to ≈15% no male inflorescences, regardless of the STS concentration used. In the books by Green (2005) and Rosenthal (2010), the authors suggest a method for making 0.3 mM STS and spraying the entire female plant until the solution drips from the plant. There is no quantitative evidence of the success of the method used. More recently, two other studies have also successfully used STS to induce male flowers: DiMatteo et al. (2020) sprayed 3 mM of STS until runoff three times at 7-day intervals after exposing the plants to short-day conditions for 12 h. Adal et al. (2021) applied 20 ml of STS (2.5 μg/ml) to whole plants on the first and third day after the start of 12-h lighting and fertilization on a foliar basis. However, these studies aimed to investigate some other aspects of male sex induction in cannabis rather than the establishment of the sex induction protocol, so no detailed data on the success of the sex reversal were presented. As far as we know, no scientific study has used colloidal silver for sex reversal in cannabis and not in other plants species. However, this method is very well known in the cannabis industry and a lot of information is available on the internet. Several other chemicals have shown alteration of cannabis sex expression, e.g., female plants treated with 75 μg of aminoethoxyvinylglycine formed only male and no intersexual flowers (Ram and Sett, 1981). Foliar spraying of male cannabis plants with 960 ppm 2-chloroethanephosphonic acid caused the highest formation of the fertile female flower (Ram and Jaiswal, 1970). A total of 100 μg/plant of cobalt chloride applied to the shoot tip triggers male sex expression in the female plants of cannabis (Ram and Sett, 1979). The mode of action of these chemicals in plants is not yet entirely deciphered. Truta et al. (2007) hypothesized that these external factors probably indirectly affect the level of endogenous auxins, which have a regulatory role on factors controlling sexual organs differentiation. The authors concluded that sex determination genes balance endogenous hormonal levels *via* signal transduction mechanism and thus enable sex reversion in sexually bipotent floral primordia. A comprehensive study of gene expression during flower development in cannabis was recently published by Adal et al. (2021), who discovered approximately 200 genes that were potentially involved in the production of male flowers in female plants. Although the exact role of all these genes was not examined further, the study opened many possibilities for further studies of the genetic background of sex expression in cannabis.

Manipulation of sex expression is of paramount importance in breeding medical cannabis, since only genetically and phenotypically female plants are used in commercial cultivation. It enables self-pollination and crossing of female plants for obtaining pure lines and feminized seeds, respectively (Ram and Sett, 1982). Upon germination, the latter produce entirely female progeny that is used for the production of female flowers. Most cannabis sex manipulation studies are performed on fiber-type hemp (Ram and Sett, 1982; Lubell and Brand, 2018; DiMatteo et al., 2020), and knowledge about the efficiency of various exogenous factors and application methods for inducing sex conversion in medical cannabis is needed.

The aim of our investigation was to test different sex manipulation methods (chemical, hormonal, and physiological) for induction of male flowers on female plants of medical cannabis and to evaluate their efficiency based on the number of male inflorescences and male flowers, by evaluation of pollen viability, germination potential *in vitro* and *in vivo*, and seed set. In addition, the selected treatment was implemented in a breeding program for crossing a population of female plants of a high CBD breeding population of medical cannabis to verify the usefulness of such treatments in the high-valued medical cannabis industry.

## Materials and Methods

### Plant Material and Growing Conditions

The experiment was carried out using plants of two breeding populations of medical cannabis, namely MX-CBD-11 and MX-CBD-707, owned by MGC Pharmaceuticals Ltd. and described in Mestinšek Mubi et al. (2020). They were grown as part of a joint research project between the Biotechnical Faculty of the University of Ljubljana and MGC Pharmaceuticals under license from the Slovenian Ministry of Health.

The mother plants (48 different genotypes of MX-CBD-707 and 31 different genotypes of MX-CBD-11) were grown from feminized seeds. Rooted cuttings were made from lateral shoots of mother plants. All plants were grown in 3.5 L pots (substrate Kekkila, Finland) in a step-in growth chamber under 24–26°C and 16/8 light/dark regime. The light was ensured by using 600-W high-pressure sodium (HPS) lamps (Phantom HPS 600W; Hydrofarm, Petaluma, CA, United States). At the vegetative stage, plants were fertilized with a mixture of vegetative fertilizer (NPK 4-1-2) and CalMag (N-Ca-Mg 2-5-2.5) + microelements in 1:1 proportion. After 31 (Experiment 1) or 60 (Experiment 2) days of vegetative growth, the plants were fertilized with a mixture of flowering fertilizer (NPK 1-3-5) and CalMag (N-Ca-Mg 2-5-2.5) + microelements in 1:1 proportion and subjected to a 12/12 photoperiod.

### Design and Performance of the Experiments

In the first experiment, eight different treatments were applied using two different growth regulators in different concentrations and modes of application, along with one physiological treatment (cut) and control (no application) (Table 1). The concentrations (amounts) of STS and GA were chosen based on literature data (Ram and Sett, 1982; Green, 2005; Rosenthal, 2010) and the variant “cut” was based on the recommendations from the growers. The experiment was designed as a randomized complete block design with two factors: seven male induction treatments and control on two breeding populations, with six replicates (potted plants) for each combination of factors.

**TABLE 1:** Treatments of the first experiment of male flowers induction on female plants of *Cannabis sativa*.

**Variant**	**Treatments on whole plants (concentration of STS)**	**Treatments on shoot tips (amount of STS)**
1	20 mM	
2	0.7 mM	
3		50 μg
4		100 μg
5		150 μg

6	Spraying whole plants with 0.01% gibberellic acid (GA3)	
7	Cutting plants to a height of two nodes	
8	Control (no application)	

**FIGURE 1:**
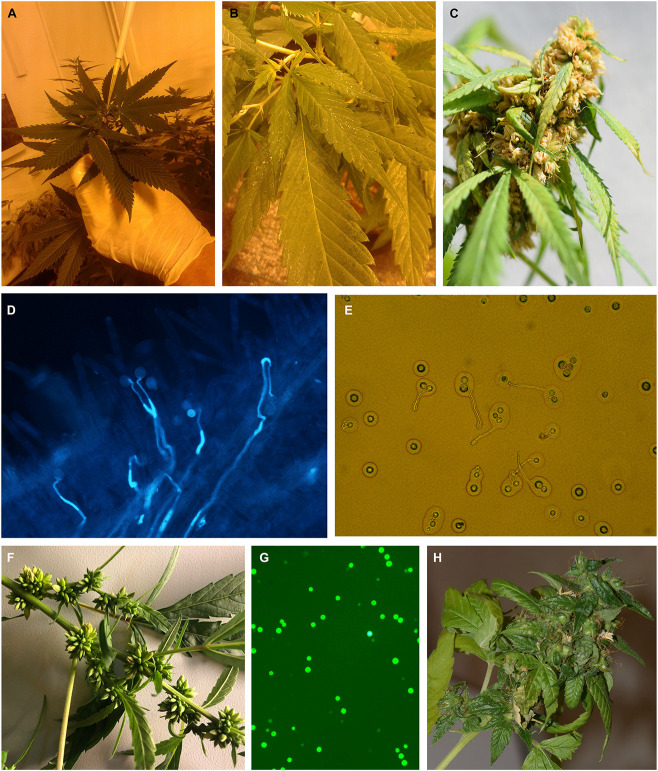
The induction of male flowers on female plants of medical cannabis. **(A)** Application of silver thiosulfate (STS) on shoot tip. **(B)** Yellow spots on the leaves 1 week after spraying with 20 mM STS. **(C)** Male inflorescence at full flowering. **(D)** *In vivo* germination of pollen. **(E)** *In vitro* germination of pollen. **(F)** The occurrence of male flowers on female plants of breeding population MX-CBD-707 after spraying with 30 ppm colloidal silver every day. **(G)** Viable pollen stained with fluorescein diacetate (FDA). **(H)** Developing seeds after pollination of a control plant.

A total of 20 mM STS was prepared by mixing 0.1 M AgNO_3_ and 0.1 M Na_2_S_2_O_3_ in a molar ratio of 1:4. 0.7 mM STS was prepared by 30x dilution of 20 mM STS with water. GA3 (Duchefa) was dissolved in double-distilled water and applied in 0.01% concentration. Spraying with STS or GA3 (treatments 1, 2, and 6) was performed once at the beginning of the experiment until runoff. For treatments 3, 4, and 5, 10 μl of STS stock solutions (1, 2, and 3 μg/μl, respectively) were applied for five consecutive days on the apical shoot tip until final amounts of STS were reached (50, 100, and 150 μg of STS, respectively) (Figure 1A). For treatment 7, plants were cut down to the height of the first two nodes. Plants from the eighth treatment represented a control group and no treatment was performed. When the treatments were applied and the experiment began, the plants (age of 31 days) were put on under a 12/12 light/dark regime to induce flowering.

In the second experiment (Table 2), 45 plants of breeding population MX-CBD-707 were treated for male sex induction. After 60 days on vegetative growth, they were first exposed to three different lighting regimes (henceforth referred to as “pretreatments;” 15 plants per pretreatment), which was followed by four different treatments: spraying whole plants with STS (Green, 2005), spraying whole plants with colloidal silver once, or every day until anthesis (recommendation of grower), and control (non-treated plants).

**TABLE 2:** Combinations of pretreatments, treatments, and growing photoperiods used in the second experiment of male flowers induction.

**Variant**	**Pretreatment**	**Treatment**	**Photoperiod after treatment (light/dark)**
9	1 week under constant light (168 h of light)	0.3 mM STS	12/12
10	1 week under constant light (168 h of light)	0.3 mM STS	96/72
11	1 week under constant light (168 h of light)	30 ppm colloidal silver once	12/12
12	1 week under constant light (168 h of light)	30 ppm colloidal silver every day until anthesis	12/12
13	1 week under constant light (168 h of light)	Control – not treated plants	12/12

14	1 week under constant dark (168 h of dark)	0.3 mM STS	12/12
15	1 week under constant dark (168 h of dark)	0.3 mM STS	96/72
16	1 week under constant dark (168 h of dark)	30 ppm colloidal silver once	12/12
17	1 week under constant dark (168 h of dark)	30 ppm colloidal silver every day until anthesis	12/12
18	1 week under constant dark (168 h of dark)	Control – not treated plants	12/12

19	1 week under 18/6 light/dark photoperiod	0.3 mM STS	12/12
20	1 week under 18/6 light/dark photoperiod	0.3 mM STS	96/72
21	1 week under 18/6 light/dark photoperiod	30 ppm colloidal silver once	12/12
22	1 week under 18/6 light/dark photoperiod	30 ppm colloidal silver every day until anthesis	12/12
23	1 week under 18/6 light/dark photoperiod	Control – not treated plants	12/12

*Each variant was applied to three plants.*

After the application of silver solutions, the plants from almost all combinations of pretreatment and treatment were exposed to a 12/12 light/dark regime to induce flowering. After application of 0.3 mM STS on whole plants, a stress-inducing photoperiod with 96 h of light and 72 h of the dark was tested and compared with the results of the same STS treatment followed by a normal 12-h photoperiod.

### Measurements of Response Variables

In the first experiment, five different response variables were analyzed, namely:

(1) Plant height.
(2) A number of nodes per plant, both expressed as a ratio between the final state (measurement/count at the end of the experiment) and the initial state (counted at the start of the experiment prior to (pre)treatments). In this way, not only the final morphology of the plants was taken into account, but also the initial state of the plants.
(3) Number of all inflorescences per plant.
(4) The number of inflorescences with one male flower or more per plant.
(5) The number of male flowers per plant. Variables were counted 31 days after the beginning of the experiment.

In the second experiment, the number of male flowers on breeding population MX-CBD-707 was counted 37 days after the beginning of the experiment.

### Viability and Germination of Pollen

Several tests were performed to verify the viability of pollen developed in induced male flowers. Pollen was stained with FDA at a final concentration of 1 μg/ml and analyzed under an epi-fluorescent microscope (Nikon Eclipse 80i) with filter sets for the detection of green fluorescence. The germination of pollen was first tested *in vitro* on solidified germination medium composed of 170 g/l sucrose, 0.1 g/l H_3_BO_3_, 0.432 g/l Ca(NO_3_)_2_^∗^4H_2_O, and pH 7.0. The Petri dishes were incubated in the dark at room temperature for 24 h and the results were detected under the microscope. Furthermore, *in vivo* germination of pollen was tested by pollinating female flowers of control, not treated, and plants. The stigmas of pollinated female flowers were collected after 24 h, stained with 1% aniline blue in 0.1 N Na_3_PO_4_, as described by Murovec and Bohanec (2013), and analyzed under the epi-fluorescent microscope with filter sets for the detection of blue fluorescence. Finally, pollen from some of the treatments was used for pollination of female control plants, and the number of developing seeds was counted 2 weeks after pollination.

### Statistical Analysis

Both sex induction experiments were performed once and analyzed as a two-factorial experiment, where, the main effect of factors and their interaction was statistically quantified using ANOVA. Before analysis, each response variable was tested for assumptions about normal distribution and homogeneity of the treatment variances by Levene’s test. In the case of non-homogeneity of variances, data were transformed to sqrt(y). Significant differences in mean values indicated by ANOVA were evaluated using Tukey’s test (α = 0.05). All statistical analyses were performed using the agricolae package in the statistical software program R version 3.2.5 (R Core Team, 2019). Data are presented as untransformed means ± SE. Graphs were drawn in the Microsoft Excel program.

### Implementation of Sex Manipulation for Breeding Medical Cannabis

In order to verify the usefulness of our approach for breeding medical cannabis, we first analyzed the cannabinoid content in inflorescences of 48 mother plants of breeding population MX-CBD-707. The high performance liquid chromatography (HPLC) analysis was performed as described by Gul et al. (2015), with modifications described in Laznik et al. (2020).

Based on the results, 23 mother plants with high total CBD (tCBD) and low total THC (tTHC) content with a ratio of tCBD:tTHC > 13 were selected for further breeding. From each selected mother plant, two clones were produced and cultured under vegetative conditions in separate chambers. One clone per mother plant was exposed to flowering conditions of light and fertilization and was sprayed with 30 ppm colloidal silver every day until the appearance of the male flower. The other clone was exposed to a flowering regime without any treatment in order to stimulate female flowering. The masculinized and non-treated plants were joined in the same flowering room upon the occurrence of male flowers on treated plants and left to cross-pollinate due to forced ventilation in the flowering chamber.

Mature seeds were collected, soaked in water for 12 h in the dark at room temperature, and then sown in polystyrene plates with 84 holes in the substrate Kekkila (Finland). The polystyrene plates were incubated at 25°C with a photoperiod of 16/8 days/nights and 60% humidity. The emerged seedlings were clonally propagated and the clones of 74 genetically different seedlings were analyzed for their cannabinoid content in inflorescences as described above. Plants from this breeding experiment were grown in the vegetative and flowering stages like the other plants in this study (described in section “Plant Material and Growing Conditions”).

## Results

### Experiment 1

#### Silver Thiosulfate Negatively Effects the Growth of Plants and Morphology

On the plants from treatment 1 (sprayed with 20 mM STS), yellow spots on the leaves were observed 1 week after application, and then the spots started to dry (Figure 1B). The plants began to lose leaves after 3 weeks of flowering. The plants from treatment 2 (sprayed with 0.7 mM STS) had fewer yellowish spots and dry leaves. Their growth and development were not as inhibited as those of plants from treatment 1.

Treatments 3, 4, and 5 (application of 50, 100, and 150 μg STS on shoot tip, respectively) also caused some physiological responses. Three weeks after application, the young leaves, which were not fully developed at the time of treatment, began to show injuries and deformations. The leaves began to dry throughout the plant, not only at the shoot tip, where STS was applied. The intensity of these injuries coincided with the amount of STS applied at the shoot tip. The higher the amount of STS applied to the shoot tip, the more severe effect it had to plant morphology and fitness. Plants from treatment 6 (sprayed with 0.01% GA) began to grow in length and intensive elongation of internodes was observed.

Male inflorescences began to appear 3 weeks after treating female plants. They were first observed in treatment 1 (20 mM STS, sprayed), followed by the appearance of male flowers on plants from treatments 2 (0.7 mM STS, sprayed), 5, 4, and 3 (application of 150, 100, and 50 μg STS on shoot tip, respectively) at intervals of 3 days as the treatments are listed. Male flowers began to open 4 weeks after treating the plants and pollen began to spread (Figure 1C). On the plants from treatments 6 (GA3), 7 (cut), and the control, only a few male flowers were observed. Plants from all treatments developed female flowers as well. No hermaphrodite flowers (i.e., pistillate flowers containing also anthers) were observed.

#### Different Treatments Induced the Formation of Male Flowers on Female Plants

The breeding population and the treatment had a statistically significant influence on the ratio of plants height, the ratio of the number of nodes, number of all inflorescences, and number of male inflorescences with only one exception (Table 3). No interaction between breeding population and treatment was found for mentioned variables.

**TABLE 3:** Influence of breeding population and treatment on the ratio of plants height, the ratio of the number of nodes, number of all inflorescences, and number of male inflorescences.

	**Height (ratio)**	**Number of nodes (ratio)**	**Number of all inflorescences**	**Number of inflorescences with male flowers**
**Breeding population (*n* = 48)**				
MX-CBD-707	3.02 ± 0.15a	4.45 ± 0.26a	94.6 ± 2.7a	20.1 ± 2.7b
MX-CBD-11	1.85 ± 0.05b	3.26 ± 0.14b	79.3 ± 3.0b	28.4 ± 3.7a
*p*	***	***	***	***
**Treatment (*n* = 12)**				
1–20 mM STS	2.46 ± 0.26a	4.05 ± 0.41a	97.3 ± 3.7a	53.8 ± 3.4a
2–0.7 mM STS	2.39 ± 0.23a	4.01 ± 0.24a	92.0 ± 4.6a	50.9 ± 4.6a
3–50 μg STS	2.38 ± 0.21a	4.19 ± 0.39a	91.34.8 ± a	38.7 ± 4.6*ab*
4–100 μg STS	2.46 ± 0.28a	4.17 ± 0.41a	94.3 ± 4.7a	24.8 ± 2.7*bc*
5–150 μg STS	2.38 ± 0.28a	4.36 ± 0.65a	94.7 ± 4.6a	21.0 ± 3.7c
6–GA3	2.78 ± 0.39a	4.09 ± 0.37a	88.3 ± 5.2*a*	2.2 ± 0.7d
7–Cut	1.82 ± 0.28a	1.84 ± 0.15b	46.8 ± 3.0b	1.7 ± 0.7d
8–Control	2.82 ± 0.27a	4.09 ± 0.38a	91.4 ± 3.7a	1.0 ± 0.4d
*p*	ns	***	***	***

Mean values are followed by SE. The ratio of height and number of nodes means a ratio between the final state (measurement/count at the end of the experiment) and the initial state (counted at the start of the experiment prior to (pre)treatments); STS, silver thiosulfate; GA3, gibberellic acid (0.01%); treatments 1, 2, and 6, spraying once with the chemical until runoff of the leaves; treatments 3, 4, and 5, application on shoot tip. Mean values followed by different letters are significantly different at the 5% level of probability (Tukey); ***p < 0.001; ns, not significant.

The statistically significant breeding populations (*p* < 0.001) differed for all four measured response variables, where MX-CBD-707 exposed more intensive morphological growth (higher ratio for height and number of nodes) and formed more inflorescences compared with MX-CBD-11. But the breeding population MX-CBD-11 exhibited a higher number of male inflorescences.

Treatment had no influence on the height ratio, despite the fact that cut plants exhibited the least growth. On the contrary, the ratio of a number of nodes significantly differed among treatments (*p* < 0.001), where cut plants showed the smallest increase in a number of nodes. The same observation goes with the number of all inflorescences, where plants from this treatment developed the least inflorescences. The differences among treatments are more pronounced for the number of inflorescences with male flowers, where spraying with 20 mM STS stimulated the development of the highest number of male inflorescences, followed by spraying with 0.7 mM STS and application of 50 μg STS on shoot tip. Lower numbers of inflorescences with male flowers with no statistical difference were observed in treatments 6 (GA3), 7 (cut), and the control.

#### The Number of Male Flowers Was Influenced by the Interaction Effect Between Both Factors

**FIGURE 2:**
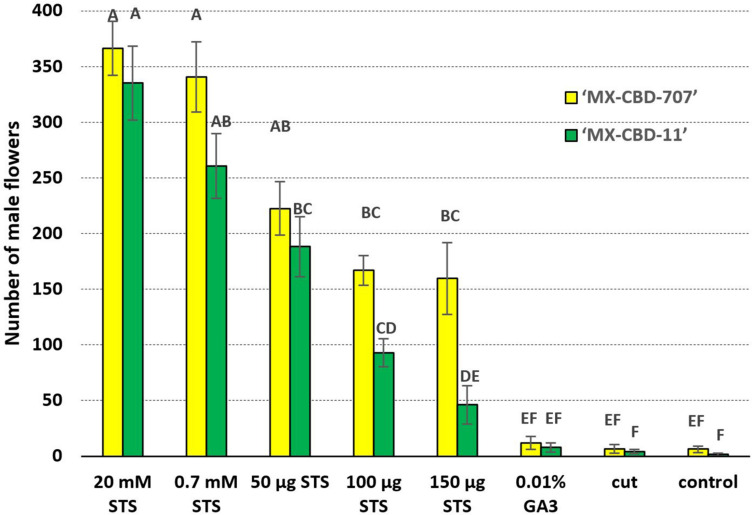
The number of induced male flowers per plant is indicated by the interaction effect between breeding population and treatment. Mean values followed by different letters are significantly different at the 5% level of probability (Tukey). Horizontal bars represent SE (± SE).

Statistically significant interaction (*p* = 0.0393) was found between main factors for the number of male flowers per plant (Figure 2). The highest number of male flowers (for both breeding populations) was observed after treatments 1 and 2 (sprayed with 20 and 0.7 mM STS), followed by treatments 3, 4, and 5 (application 50, 100, and 150 μg STS on shoot tip, respectively). The last three treatments (6 – GA3, 7 – cut, and control) produced a significantly lower number of male flowers. In all the eight tested treatments, the breeding population MX-CBD-11 developed a higher number of male flowers compared with MX-CBD-707 (Figure 2).

### Experiment 2

#### Colloidal Silver Induced Formation of Fertile Male Flowers on Female Plants

In the second experiment, the effect of different pretreatments (168 h light, 168 h dark, and alternation of 18/6 light/dark) before application of 0.3 mM STS or 30 ppm colloidal silver was studied. After spraying the whole plants, they were exposed to a constant 12/12 light/dark photoperiod or to a stress-inducing photoperiod (one treatment). Pretreatment, as well as treatment, had a statistically significant influence on the number of male flowers, but their interaction was not observed (Table 4). The highest average number of male flowers per plant (339) was achieved after pretreating plants at the usual light regime for vegetative growth (18 h of light and 6 h of darkness), while incubation in darkness for 168 h caused the lowest appearance of male flowers. Among the tested treatments, 0.3 mM STS caused the highest formation of male flowers, followed by the same treatment and exposing plants to stress-inducing light regimes. In contrast to spraying of 0.3 mM STS, which induces male flowering after only one application, the colloidal silver had to be sprayed every day until the formation of male flowers and yielded on an average 293 male flowers per plant. Spraying plants with colloidal silver only once produced a negligible number of male flowers, the results being practically equal to the results of control plants, which were not sprayed with any silver solutions (Tables 4, 5). The appearance of induced male flowers and the viability of pollen are shown in Figures 1F,G.

**TABLE 4:** Influence of pretreatment and treatment on a number of male flowers per plant of MX-CBD-707.

	**Number of male flowers per plant**
**Pretreatment (*n* = 15)**	
18/6 light/dark	339 ± 87a
168 h light	253 ± 70ab
168 h dark	155 ± 62b
*p*	*
**Treatment (*n* = 9)**	
1–0.3 mM STS, 12/12	497 ± 60a
2–0.3 mM STS, 96/72	448 ± 99a
3–30 ppm colloidal silver every day, 12/12	293 ± 91a
4–30 ppm colloidal silver once, 12/12	1 ± 1b
5–Control, 12/12	0 ± 0b
*p*	***

Mean values are followed by SE. Pretreatments: 18/6 light/dark, incubation of plants 1 week under 18/6 light/dark photoperiod; 168 h light, incubation of plants 1 week under constant light; 168 h dark–incubation of plants 1 week under constant dark. Treatment denotes the use of silver solutions and control treatment (non-treated plants); treatments 1, 2, and 4, spraying once with the chemical until runoff of the leaves; treatment 3, spraying with the chemical until runoff of the leaves every day until anthesis. Photoperiod after treatment: 12/12, exposure to 12/12 light/dark regime; 96/72, exposure to photoperiod with 96 h of light and 72 h of dark. STS–silver thiosulfate. Mean values followed by different letters are significantly different at the 5% level of probability (Tukey); ***p < 0.001; *p < 0.05.

**TABLE 5:** Number of male flowers per plant and number of germinated pollen cells *in vitro* after the induction of male flowering on female plants of MX-CBD-707.

**Pretreatment**	**Treatment**	**Average number of male flowers per plant ± SE**	**Percentage of pollen cells germinated *in vitro* ± SE**
168 h light	0.3 mM STS, 12/12	577 ± 89	3.48 ± 0.99
168 h dark	0.3 mM STS, 12/12	431 ± 160	2.40 ± 1.48
18/6 light/dark	0.3 mM STS, 12/12	482 ± 66	7.18 ± 3.02

168 h light	0.3 mM STS, changing 96/72	497 ± 82	0.00
168 h dark	0.3 mM STS, changing 96/72	129 ± 11	0.00
18/6 light/dark	0.3 mM STS, changing 96/72	717 ± 100	0.00

168 h light	30 ppm colloidal silver once, 12/12	0	/
168 h dark	30 ppm colloidal silver once, 12/12	0	/
18/6 light/dark	30 ppm colloidal silver once, 12/12	0	/

168 h light	30 ppm colloidal silver every day, 12/12	285 ± 59	0.91 ± 0.91
168 h dark	30 ppm colloidal silver every day, 12/12	216 ± 182	2.66 ± 1.11
18/6 light/dark	30 ppm colloidal silver every day, 12/12	379 ± 190	4.34 ± 1.62

168 h light	−, 12/12	0	/
168 h dark	−, 12/12	0	/
18/6 light/dark	−, 12/12	0	/

*Pretreatments: 18/6 light/dark, incubation of plants 1 week under 18/6 light/dark photoperiod; 168 h light, incubation of plants 1 week under constant light; 168 h dark, incubation of plants 1 week under constant dark. Treatment denotes the use of silver solutions and control treatment (non-treated plants); treatments 1, 2, and 4, spraying once with the chemical until runoff of the leaves; treatment 3, spraying with the chemical until runoff of the leaves every day until anthesis. Photoperiod after treatment: 12/12, exposure to 12/12 light/dark regime; 96/72, exposure to photoperiod with 96 h of light and 72 h of dark. STS, silver thiosulfate.*

#### Pollen Successfully Germinated *in vitro* and *in vivo*

The *in vitro* germination test showed that the induced male flowers produced viable pollen that is able to germinate *in vitro* on solidified germination medium (Figure 1E and Table 5).

The germination ability of pollen was confirmed also with *in vivo* pollination of female flowers. After 24 h, the germinating pollen tubes were clearly visible on stigmas stained with aniline blue (Figure 1D).

In order to verify the ability of pollen to fertilize female flowers and produce feminized seeds, the pollen was collected from treated plants of breeding population MX-CBD-707 and used for pollination of different shoots of one control (non-treated) plant. Two weeks after pollination, the number of developing feminized seeds was counted, which is presented in Table 6 and Figure 1H.

**TABLE 6:** Number of developing seeds 2 weeks after pollination with pollen from plants MX-CBD-707 induced with different light (pre)treatments and silver applications.

**Pretreatment**	**Treatment**	**No. of developing seeds**
168 h light		12
168 h dark	0.3 mM STS, 12/12	7
18/6 light/dark		18

168 h light		12
168 h dark	0.3 mM STS, 96/72	3
18/6 light/dark		1

168 h light		39
168 h dark	30 ppm colloidal silver every day, 12/12	31
18/6 light/dark		11

*Pretreatments: 18/6 light/dark, incubation of plants 1 week under 18/6 light/dark photoperiod; 168 h light, incubation of plants 1 week under constant light; 168 h dark, incubation of plants 1 week under constant dark. Treatment denotes the use of silver solutions and control treatment (non-treated plants); treatments 1, 2, and 4, spraying once with the chemical until runoff of the leaves; treatment 3, spraying with the chemical until runoff of the leaves every day until anthesis. Photoperiod after treatment: 12/12, exposure to 12/12 light/dark regime; 96/72, exposure to photoperiod with 96 h of light and 72 h of dark. STS, silver thiosulfate.*

### Breeding MX-CBD-707

Analysis of cannabinoid profile revealed that 48 plants of MX-CBD-707 contained between 3.47 and 11.70% and 0.41 to 9.91% of tCBD and tTHC, respectively. The ratios between tCBD and tTHC thus varied between 0.9 and 18.8, which represents an almost 21 fold difference. In order to stabilize CBD extraction from female flowers, we selected 23 plants with tCBD to tTHC ratios above 13.

**FIGURE 3:**
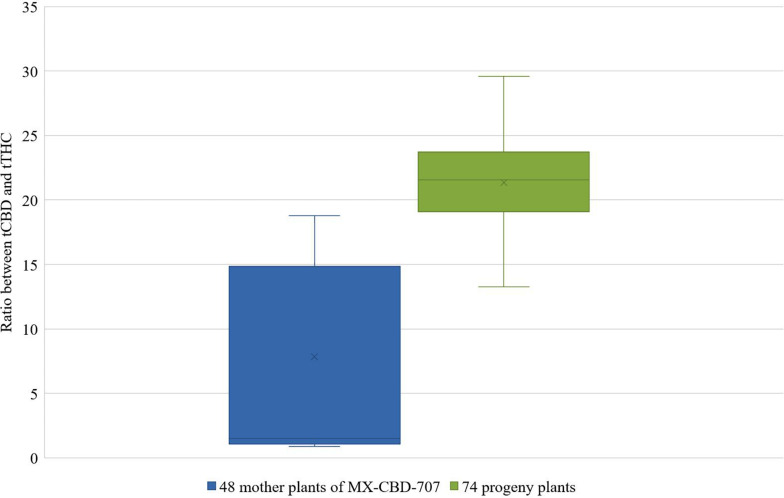
Distribution of the ratios between tCBD and tTHC in 48 mother plants of breeding population MX-CBD-707 (left-blue) and 74 of their progeny (right-green).

These mother plants were cloned, induced to produce male flowers by spraying them with 30 ppm colloidal silver every day, and left to cross-pollinate in a contained flowering chamber. The colloidal silver treatment was chosen based on our results obtained in the above described experiments, which demonstrated the best performance in terms of the number of *in vitro* germinated pollen grains and of the number of developing seeds (Tables 5, 6). Seeds were left on plants until maturity when they were sown, and a 64.3% germination rate was recorded. The cannabinoid analysis of 74 seedlings showed that their flowers contained from 1.77 to 24.34% and 0.09 to 0.85% of tCBD and tTHC, respectively. The ratio between tCBD and tTHC varied between 13.26 and 29.58 (Figure 3).

## Discussion

Alteration of the reproduction system in cannabis, in the form of the appearance of male flowers on female plants, is a useful phenomenon in cannabis breeding. It enables self-pollination and/or crossing plants that are genetically female. Moreover, it leads to offspring seeds that are entirely feminized. As such, they are highly valuable in medical cannabis production, which relies exclusively on phenotypically female plants (Soler et al., 2017).

Ethylene is a known gaseous plant hormone, which is involved in sex expression in plants. It promotes femaleness and inhibitors of ethylene biosynthesis or ethylene response suppress the development of female reproductive organs, thus promoting masculinity (Kumar et al., 2009). The mode of action was partly elucidated recently in cucumber and melon, where, Tao et al. (2018) demonstrated that ethylene signaling is directly involved in interaction among sex determination-related genes by controlling ethylene-responsive transcription factors CsERF110 and CmERF110 (Tao et al., 2018). Silver ions from STS and colloidal silver act as ethylene antagonists, thus blocking its function, and in this way probably enable male sex induction (Ram and Sett, 1982). Ram and Sett (1982) showed for the first time that STS is capable of male sex induction on wild accession of *C. sativa* L. They also discovered that STS was more efficient compared to AgNO_3_, probably due to the faster transport of STS through plants. Recently, Adal et al. (2021) used STS for the chemical induction of male flowers on female plants. They identified over 10,500 differentially expressed genes, of which, around 200 are potentially responsible for male flower development on female plants. Their study confirmed that sex determination in cannabis flowers is controlled primarily at the genetic level. However, the expressed genes appeared to be involved in several pathways, such as phytohormone signaling, floral development, metabolism of lipids, sugar, and others, implying that the process of sex expression in cannabis plants occurs at multiple levels.

In our experiment, 23 different treatments (chemical using STS or colloidal silver; hormonal using GA; and physiological by intensive cutting) were used for induction of male flowers on female plants, in order to evaluate their influence on sex expression in medical cannabis. Plant height and number of nodes, which assessed the influence of treatments on plant growth, showed no negative influence as a result of our treatments. Although Ram and Sett (1982) reported that after application of STS on shoot tips the treated plants became black, the young leaves became decolorized, wilted, and deformed, etc., less pronounced effects of STS on plant growth and morphology were observed in our study. Even the highest amount of STS added on the shoot tip in our research (150 μg) had no negative influence on plant growth (Table 3), while application of 100 μg on the shoot tip in the study of Ram and Sett (1982) caused a total collapse of the shoot tip, decreased leaf area, and reduced plant growth in height after treatment. The number of male flowers per plant in our study was similar to the number they counted for the same treatment (application of 100 μg; up to 110 male flowers), but our research also showed that applying STS to whole plants is more efficient than the application of STS to the shoot tips.

The interaction effect between genotype and treatment on a number of male flowers in this study proved that genotype affects the success of male flower induction. Overall, we observed a higher number of male flowers developed per plant compared with the study of Ram and Sett (1982), who treated fiber-type hemp plants; while in our experiment, plants of medical cannabis were used. Besides, we applied STS by spraying whole plants, where Ram and Sett (1982) add STS to shoot tip only and this could also be the reason for the obtained variation. Differences in maleness induction were also observed by comparing our results with Lubell and Brand (2018), who used STS for the induction of male flowers on female hemp plants. Although the number of male flowers in their study was not exactly counted, they determined up to ≈85% of inflorescences with male flowers, where in our study, only approximately 55% of inflorescences contained male flowers. Lubell and Brand (2018) found that one (out of four tested) hemp strain was more prone to sex conversion and exhibited a higher level of masculinization. The phenomenon of genotype dependency on sex induction was also observed by Moliterni et al. (2004), who noticed that European hemp varieties exhibit different stages of resistance to sex reversion treatments.

The assumption about genotype specificity for sex reversion was confirmed by the results of our first experiment, in which breeding population MX-CBD-11 outperformed MX-CBD-707 in terms of male flower production after all seven different treatments. Since the treatments were identical for both breeding populations and performed simultaneously under the same flowering conditions, the results clearly demonstrate genotype dependency of physio-morphologic response to silver compounds.

Comparison of pollen from naturally male hemp plants and masculinized female ones showed that the latter produce a significantly higher number of irregular or misshapen pollen grains that are inefficient in dispersal from anthers and had a lower germination rate (DiMatteo et al., 2020). In our study, a comparison between pollen from masculinized female plants and male plants was not possible, because the medical breeding populations contained exclusively female plants. We, therefore, decided to test pollen viability and its germinability *in vitro* and *in vivo* and, finally, evaluated seed set after pollination of female plants with pollen from masculinized plants. Our results (Figure 1 and Tables 5, 6) confirmed the viability and functionality of the pollen developed on masculinized female plants. It demonstrated that colloidal silver is also very efficient for the induction of male flowers (as reported in Table 5), and this is the first report about the induction of male flowers on female plants of cannabis with colloidal silver.

On the other hand, spraying with hormone GA3 had no significant effects on male flower induction in our medical cannabis plants, as was also shown by Sarath and Ram (1978). Although, Chailakhyan (1979) demonstrated that GA3 has a strong effect on the appearance of male flowers, they applied the hormone through roots of very young cannabis plants, which is impractical for breeding purposes. In addition to chemical and hormonal triggers, physiological stress caused by mechanical damage was also expected to increase the likelihood of sex reversion (Clarke, 1999). However, intensive cut, which was one of our treatments, did not produce positive results in terms of induction of male flowers.

Some male flowers appeared on the control plants, where no staminate flowers were expected. It is possible that unwanted drift of STS from treated plants to the control plants occurred in the growing room since strong ventilation was used during growth. On the other hand, it has been recently shown by Punja and Holmes (2020) that male flowers can form spontaneously on up to 10% of female plants, therefore, the possibility of unwanted spontaneous sex conversion cannot be excluded.

Finally, the protocol using colloidal silver was successfully used for breeding female plants of medical cannabis breeding population MX-CBD-707. Entirely, the female progeny was obtained after crossing the parental population of females induced to form male flowers, thus confirming the theory of producing exclusively feminized seeds when crossing only XX plants (Green, 2005). Furthermore, a significant improvement of tCBD/tTHC ratio was observed (with a maximal tCBD content of 29.58% measured in a plant with a tTHC as low as 0.82%) after crossing only 23 selected parental plants. It shows that crossing plants selected based on their chemotype profile improves the genetic constitution of the breeding population and consequently enables the development of new varieties with improved cannabinoid profiles.

## Conclusion

To the best of our knowledge, this is the first comprehensive scientific report on the induction of fertile male flowers on female plants of medical cannabis (*Cannabis sativa* L.). Previous reports on gender manipulation in cannabis were performed on fiber-hemp genotypes (Ram and Sett, 1982; Lubell and Brand, 2018; DiMatteo et al., 2020), while methods for medical cannabis have been shared among growers for years, but the information was not obtained in a manner based on scientific methodologies.

## Data Availability Statement

The original contributions presented in the study are included in the article/supplementary material, further inquiries can be directed to the corresponding author.

## Author Contributions

MF and JM conceived and designed the study and wrote the manuscript. MS performed the experiments. MF, MS, and JM analyzed the data. All authors read and approved the manuscript.

## Conflict of Interest

The authors declare that the research was conducted in the absence of any commercial or financial relationships that could be construed as a potential conflict of interest.

## Publisher’s Note

All claims expressed in this article are solely those of the authors and do not necessarily represent those of their affiliated organizations, or those of the publisher, the editors and the reviewers. Any product that may be evaluated in this article, or claim that may be made by its manufacturer, is not guaranteed or endorsed by the publisher.
